# Cognitive Performance of Patients with Adult 5q-Spinal Muscular Atrophy and with Amyotrophic Lateral Sclerosis

**DOI:** 10.3390/brainsci11010008

**Published:** 2020-12-23

**Authors:** Alma Osmanovic, Gary Wieselmann, Lucas Mix, Hannah Alexandra Siegler, Mareike Kumpe, Gresa Ranxha, Claudia D. Wurster, Alexander Steinke, Albert C. Ludolph, Bruno Kopp, Dorothée Lulé, Susanne Petri, Olivia Schreiber-Katz

**Affiliations:** 1 Department of Neurology, Hannover Medical School, 30625 Hannover, Germany; Osmanovic.Alma@mh-hannover.de (A.O.); Gary.Wieselmann@stud.mh-hannover.de (G.W.); Siegler.Hannah@mh-hannover.de (H.A.S.); Kumpe.Mareike@mh-hannover.de (M.K.); Gresa.Ranxha@stud.mh-hannover.de (G.R.); Steinke.Alexander@mh-hannover.de (A.S.); Kopp.Bruno@mh-hannover.de (B.K.); Petri.Susanne@mh-hannover.de (S.P.); 2 Department of Neurology, Neuropsychology, University of Ulm, 89081 Ulm, Germany; lucas.mix@t-online.de (L.M.); albert.ludolph@rku.de (A.C.L.); dorothee.lule@uni-ulm.de (D.L.); 3 Department of Neurology, University of Ulm, 89081 Ulm, Germany; claudia.wurster@uni-ulm.de; 4 Deutsches Zentrum für Neurodegenerative Erkrankungen (DZNE), 89081 Ulm, Germany

**Keywords:** motor neuron disease, spinal muscular atrophy (SMA), amyotrophic lateral sclerosis (ALS), Edinburgh Cognitive and Behavioural ALS Screen (ECAS), German vocabulary test (Wortschatztest, WST), cognitive function

## Abstract

Motor neuron diseases, such as spinal muscular atrophy (SMA) and amyotrophic lateral sclerosis (ALS), share several clinical similarities while differing substantially in etiology, disease onset and progression. Cognitive dysfunction, a clinically relevant non-motor feature in a substantial proportion of ALS patients, has been less frequently investigated in SMA. In this prospective multicenter cross-sectional study, cognitive function was assessed by the Edinburgh Cognitive (and Behavioural) ALS Screen (ECAS) and a German vocabulary test (Wortschatztest, WST) in 34 adult patients with SMA types 2–4 and in 34 patients with ALS. Demographic and clinical parameters were assessed to identify factors that potentially influence cognitive function. While SMA and ALS patients were comparable in the vocabulary test, on average, SMA patients performed better than ALS patients in the cognitive domains of memory, language and executive function. Better cognitive abilities in SMA patients seemed to be related to the early onset, rather than the extent or the duration, of their physical handicap. Future studies should focus on disease-specific cognitive functions in SMA.

## 1. Introduction

Degenerative motor neuron diseases are primarily characterized by the loss of motor neurons leading to progressive muscle weakness, atrophy and ultimately death often due to respiratory insufficiency. Spinal muscular atrophy (SMA), an autosomal recessively inherited motor neuron disease caused by mutations in the *survival of motor neuron 1* (*SMN1*) gene, is differentiated into four main subtypes on the basis of age at onset and best motor function achieved [1,2]. While type 1 represents the most severe infantile phenotype, type 2 patients, characterized by onset between seven and eighteen months of age, achieve the ability to sit unsupported, but not to walk independently. SMA type 3 includes clinically heterogeneous patients, who typically learn to walk independently, but may lose this ability again over the course of the disease. The mildest SMA type 4 phenotype manifests predominantly in adulthood but may also show a juvenile onset in some cases [2,3]. Amyotrophic lateral sclerosis (ALS), the most common adult-onset motor neuron disease, is characterized by the rapidly progressive clinical correlates of the loss of cortical, brain stem and spinal motor neurons. On average, death due to respiratory insufficiency occurs three to five years after symptom onset [4].

Although SMA and ALS are both motor neuron diseases, patients differ regarding age at onset, duration of illness and speed of functional decline. ALS starts in late adulthood and typically swiftly leads to severe physical impairment with paresis up to tetraplegia, nutritional and respiratory insufficiency. Recent evidence, however, suggests that both diseases share common pathomechanisms such as neuroinflammation, intrinsic muscle defects, metabolic perturbations, selective motoneuron vulnerability, defects in neuron excitability and immune organ dysfunction [5].

Non-motor neuron symptoms have been identified more frequently in ALS than in SMA [6], among them cognitive and behavioral changes ranging from slight impairment to manifest frontotemporal dementia in ALS [7,8,9,10]. Cognitive function has not been studied extensively in adult SMA patients yet [11,12,13]. Newly developed therapeutic agents, such as nusinersen, risdiplam or onasemnogene abeparvovec with the potential to change the natural history of the disease fundamentally, render this issue even more relevant [14,15,16].

Expression of SMN protein has been detected in the human forebrain by immunochemistry, and therefore, a role during brain development is assumed [17]. Few studies on cognition in SMA, mainly in children, showed different results. In comparison to published normative data and to the similarly physically disabling disease Duchenne muscular dystrophy (DMD), no significant differences in intellectual performance of SMA patients were reported [18,19]. In contrast, other studies demonstrated superior cognitive abilities of SMA children and adolescents compared to those with DMD [20,21,22,23]. Yet another study investigated cognitive functions in a large group of SMA children and adolescents compared to age-matched healthy controls. While the ”general” intelligence of SMA children seemed to lie in the average range, it has been hypothesized that environmentally mediated and education-dependent aspects of intelligence (e.g., (exempli gratia), crystalized aspects of intelligence) might be higher in patients with SMA. The author of the cited study assumed that children with SMA need the time until adolescence to develop effective strategies to compensate for their physical handicaps through increased acquisition of cognitive skills and knowledge [24].

In a previous study of this working group, no significant difference in cognitive function between SMA patients and healthy controls, but an influence of physical function on executive functions was observed (in press) [25]. Therefore, in this study, we aimed to further investigate the hypothesis that onset of motor deficits in early childhood results in higher education and better cognitive performance in comparison to equally physically impaired patients with later age at onset of a comparatively disabling disease, here ALS. Further, we aimed to examine whether this cognitive compensation even increases during disease progression in adulthood.

## 2. Materials and Methods

### 2.1. Study Design, Setting and Participants

This study was conducted in a prospective, non-interventional, multicenter and cross-sectional design in the neuromuscular divisions of two German universities (Hannover Medical School, MHH, and University of Ulm). SMA patients (*n* = 51) with genetically confirmed mutations in the *SMN1* gene were screened between 16 May 2017 and 15 October 2019 at both sites and ALS patients (*n* = 67) between 23 April 2015 and 13 June 2018 at MHH only. All enrolled SMA patients received therapy with the antisense oligonucleotide nusinersen [26,27]. Inclusion criteria for all patients were age ≥ 18 years and ability to perform cognitive function testing in a structured personal interview. Diagnosis of ALS was based on El Escorial diagnostic criteria (possible, probable and, laboratory supported, definite ALS) [28]. Exclusion criteria were comorbidities with an effect on cognitive performance in order to minimize influencing co-effects such as major depressive disorder as well as clinically manifest frontotemporal dementia prior to testing.

Of 51 prospectively examined SMA patients, 11 were ineligible for this study due to age <18 years. An additional five patients were not able to perform cognitive function tests (*n* = 2 due to language barrier, *n* = 1 declined testing, *n* = 2 unknown), which resulted in a number of 35 SMA patients. Due to exclusion of one further patient with complex comorbidities (major depression, epilepsy, mutism and autistic characteristics) with effects on the cognitive profile, a final number of *n* = 34 SMA patient data sets were analyzed in this study.

Out of the 67 retrospectively analyzed ALS patients, 21 were ineligible due to incomplete data, and two ALS patients were excluded due to comorbidities with effects on cognitive function (*n* = 1 due to alcohol abuse, *n* = 1 due to beginning dementia). This led to a number of *n* = 44 ALS patients. For a better comparability of results, groups were matched regarding gender and physical function, using the Amyotrophic Lateral Sclerosis Functional Rating Scale revised form (ALSFRS-R) scores. On the basis of these criteria, a further 12 patients were excluded in the matching process, resulting in a final number of *n* = 34 ALS patients. Although being a potential confounder, matching according to age was not applicable due to major differences in the age structure of both groups caused by the natural disease characteristics.

The motor impairment in both cohorts was evaluated using the ALSFRS-R [29]. The ALSFRS-R addresses different motor functions associated with activities of daily living, which are rated on a scale from 0 (no function) to 4 (complete independence of the patient). Its four categories are bulbar function (e.g., speech and swallowing), fine motor function (e.g., use of hands) and gross motor function (e.g., walking and climbing stairs) as well as respiratory function (e.g., dyspnea, ventilation assistance) with a maximal sum score of 48. Moreover, main demographics, such as gender, age at enrolment, age at disease onset, years of education and educational level, as well as disease duration, ambulatory state and care level were documented. The care levels (level 1–5) correspond to a classification according to the German health care system, with higher levels indicating a greater loss of autonomy and a need for more individual support (also see legend of Table 1).

### 2.2. Measures of Cognitive Performance

Cognitive performance was evaluated with different tests: the German version of the Edinburgh Cognitive and Behavioural ALS Screen (ECAS) in both SMA and ALS patients, and the German “Wortschatztest” (WST) in SMA and a subgroup of ALS patients.

The ECAS has been specifically designed for ALS patients, with the aim of minimizing the effect of physical disability on cognitive performance measures [33,34]. In total, ECAS consists of 16 individual tasks that can be summarized into five cognitive domains: verbal fluency (Free-words beginning with the letter S and Restrained-words beginning with the letter G but with only four letters, score 0–24); language (Naming, Comprehension, Spelling, score 0–28); executive function (Reverse Digit Span, Alternation, Inhibitory Sentence Completion, Social Cognition, score 0–48), memory (Immediate Recall, Delayed Recall, Delayed Recognition, score 0–24) and visuospatial function (Dot Counting, Cube Counting, Number Location, score 0–12). As some tasks evaluate ALS-specific cognitive impairment (executive function, language and verbal fluency, score 0–100), others test ALS-nonspecific cognitive function, subsumed as the ALS-nonspecific subdomain (memory and visuospatial function, score 0–36). Ultimately, a total ECAS score, with a maximal score of 136 (= best cognitive performance), was calculated. As cognitive abilities depend on age and years of education, specific thresholds/cut-off values have previously been defined for ALS-specific and ALS-nonspecific pathological outcomes [35]. The same cut-off values were also applied to SMA patients in this study, as no validated SMA-specific ECAS thresholds are available. As the ECAS has specifically been developed for ALS patients, its usefulness in SMA has not been demonstrated to date. However, as it excludes the effect of physical disability on cognitive measurements and allows a distinct assessment of different cognitive qualities, it appears to be useful for cognitive profiling in SMA patients as well.

The WST vocabulary test is a German test for evaluating age-independent crystalized aspects of verbal intelligence [36]. Wechsler based the quality of the vocabulary test as an intelligence test on the fact that the number of words a person has at his disposal is a measure of his ability to learn, his stock of linguistic knowledge and his general scope of imagination [37]. Impairment of word recognition and reading, the skills required in the WST, are less frequent and may only occur in advanced stages in neurodegenerative conditions [36] so that one can expect a test performance without too strong inference of disease pathology. The WST comprises 42 items, which are presented in ascending difficulty levels. Each item consists of six words, among which the one actually existing word has to be identified. During analysis, each line is checked to ensure that no more than one word in the line is crossed out. If this is the case, it is considered an error, even if the target word is among the words crossed out. Then the number of correctly solved items is added up to obtain the total score (maximal 42).

### 2.3. Statistical Analysis

Statistical analysis was performed using IBM^®^ Statistical Software Package of Social Science (SPSS^®^, Chicago, IL, USA) version 25.0. Descriptive statistics were calculated and depicted as percentage, mean and standard deviation as well as median and range. We tested all outcome scores for normal distribution by means of Shapiro–Wilk tests. We tested for equality of variances by means of Levene’s test. Differences between demographic and disease characteristics of both groups were analyzed and comparisons between clinical characteristics and outcome scores (ECAS, WST) between SMA and ALS patients were carried out by using the *t*-test (for normally distributed outcome scores when equality of variances was assumed), the Welch *t*-test (for normally distributed outcome scores when inequality of variances was assumed) or the Mann–Whitney test (for all other outcome scores). Differences in pathological scores below cut-off were analyzed by Chi-squared Test or Fisher’s Exact Test. Correlation analyses were performed with Pearson’s correlation coefficient for metric variables or Spearman’s rank correlation coefficient for ordinal variables. To examine whether demographic and clinical features contributed to the presence of cognitive impairment, multiple linear regression analysis was performed. For each cognitive domain of the ECAS (dependent variable), the significantly associated parameters such as disease (ALS or SMA), age at onset, disease duration, educational years and age at enrolment were defined as independent variables and further examined.

All *p*-values were two-tailed; a *p*-value of ≤0.05 was considered statistically significant. As this is an exploratory study, we did not correct the significance level for multiple comparisons. We reported odds ratios (OR) and point biserial correlation coefficients (r) as measures of effect sizes [38]. We interpreted effect sizes of *r* = 0.10, *r* = 0.30 and *r* = 0.50 as thresholds for small, medium and large effects [30], respectively.

This study report was structured following the reporting guidelines to Strengthening the Reporting of Observational Studies in Epidemiology (STROBE) [39].

## 3. Results

### 3.1. Patient Characterization

Disease subgroups were matched regarding gender and ALSFRS-R scores in order to obtain a comparable distribution of motor disability in both cohorts. *n* = 34 SMA and *n* = 34 ALS patients were included into the final data analysis.

The 34 adult SMA patients (eleven females and 23 males) had a mean age of 40.2 (standard deviation (SD = 13.1) years and a mean disease duration of 33.8 (SD = 13.2) years. They were compared to 34 ALS patients (13 females and 21 males) with a mean age of 65.8 (SD = 9.4) years and a mean disease duration of 2.2 (SD = 3.2) years (Table 1). Within the SMA study cohort, 14 of the patients were classified as SMA type 2, 19 patients as SMA type 3 and 1 patient as SMA type 4, who was further evaluated together with SMA type 3 patients. The ALSFRS-R mean scores were 30.8 (SD = 10.8, *n* = 34) in SMA and 31.3 (SD = 7.0, *n* = 29) in ALS patients. Ambulatory status and disability level still differed between both groups due to the differences in disease duration and progression rates. For clinical characteristics, such as age, gender and education, both cohorts were representative according to previous studies, with the exception of gender distribution in SMA patients [7,40,41,42,43].

**Figure 1:**
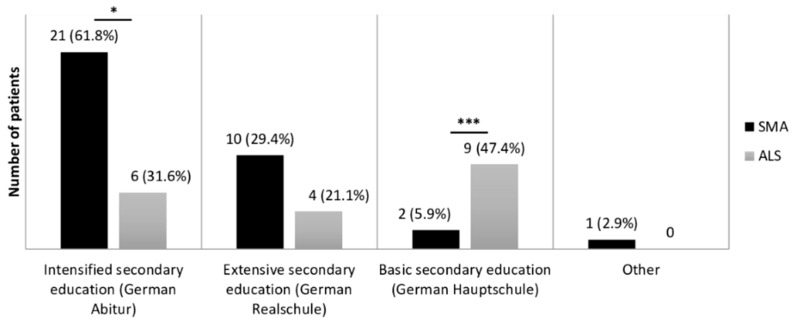
Comparison of education levels in SMA and ALS patients. Education levels, according to the German education system, were compared between SMA (*n* = 34) and ALS (*n* = 19) patients. Significance levels: * *p* ≤ 0.05, *** *p* ≤ 0.001. Abbreviations: SMA, Spinal Muscular Atrophy; ALS, Amyotrophic Lateral Sclerosis.

We found a significant difference in years of education and educational degree between both disease groups (Figure 1). SMA patients showed a mean of 16.3 (SD = 3.5, *n* = 34) years of education, while the mean of education years lay at 13.1 (SD = 2.8, *n* = 34) in ALS patients (U = 272.0; Z = −3.762; *p* < 0.001, *r* = 0.46).

Vocational activities also differed between both groups. While SMA patients mainly worked in jobs with low physical demands, e.g., jobs in information technology, law or physics, ALS patients more frequently had handicraft professions in the past.

### 3.2. Cognitive Performance of SMA Patients

Apart from the verbal fluency ECAS subdomain (*n* = 33, because one patient interrupted the test), all cognitive (sub-)scores were assessed in all SMA patients. The level of education in SMA patients was much higher in comparison to the German population. In 2017, 31.9% of German citizens had an Intensified secondary education (German Abitur) [44], in contrast to 61.8% in our group of SMA patients (*n* = 21). For further analyses, we decided to dichotomize on the basis of their education.

Thus, we separated the SMA cohort into patients with 16.5 educational years or more (*n* = 16) and patients with less than 16.5 educational years (*n* = 18) (as the mean value was 16.3 years, and approximately equals the time needed for finishing school and a German master’s degree). An influence of educational time on cognitive performance was identified for the visuospatial (mean (lower education) 11.5 vs. (versus) (higher education) 11.9; U = 96.0; Z = −2.238; *p* = 0.025, *r* = 0.38), language (mean 27.0 vs. 27.6; U = 90.5; Z = −2.039; *p* = 0.041, *r* = 0.35) and executive subdomains of the ECAS (mean 38.6 vs. 40.9; U = 77.5; Z = −2.312; *p* = 0.021, *r* = 0.40) as well as the ALS-specific domain (mean 84.7 vs. 87.6; U = 86.5; Z = −1.992; *p* = 0.046, *r* = 0.34).

No significant differences between the group with higher and lower education could be detected for the ECAS total score (mean 113.9 vs. 116.8; T = −1.220, df = 32, *p* = 0.231, *r* = 0.21), the memory (mean 17.7 vs. 17.2; T = 0.391, df = 32, *p* = 0.699, *r* = 0.07) and the verbal fluency (mean 19.1 vs. 20.4; U = 113.5; Z = −0.839; *p* = 0.401, *r* = 0.15) subdomain of the ECAS and its ALS-nonspecific domain (mean 29.2 vs. 28.5; T = 0.543, df = 32, *p* = 0.591, *r* = 0.10). Consistent with the aim of the test to measure acquired knowledge, the mean WST score was significantly higher in the more educated group of SMA patients (mean 28.7 vs. 34.2; U = 24.0; Z = −4.161; *p* < 0.001, *r* = 0.71). Further analyses did not reveal any differences in education or cognitive performance depending on ambulatory state (ambulatory *n* = 9 vs. non-ambulatory *n* = 25) or age at disease onset (<6 *n* = 21 vs. ≥6 years *n* = 13).

### 3.3. Comparison of Cognitive Performance between SMA and ALS

**Figure 2:**
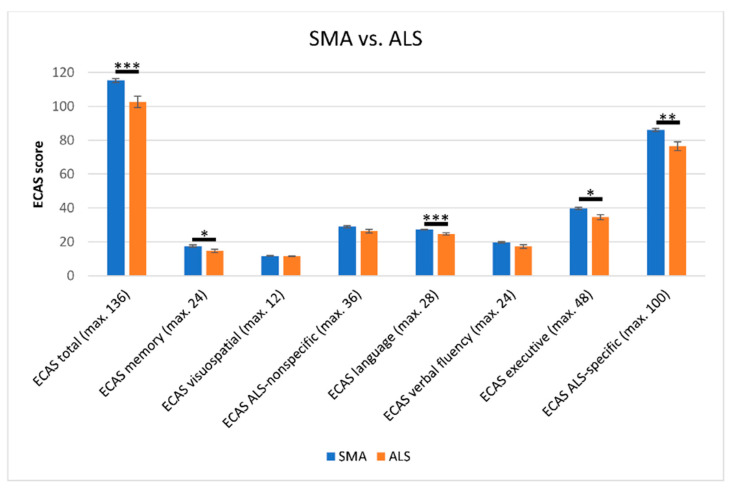
Comparison of cognitive scores according to the Edinburgh Cognitive and Behavioural ALS Screen (ECAS) in SMA and ALS patients. SMA patients scored significantly higher in the ECAS in total score, as well as in the subdomains of memory, language, executive and the ECAS ALS-specific subscores. No significant differences between both groups were detected in the ECAS visuospatial (mean 11.7 vs. 11.5; U = 512.0, Z = 1.016, *p* = 0.310, *r* = 0.12) and verbal fluency subdomains (mean 19.7 vs. 17.2; U = 431.0, Z = 1.684, *p* = 0.092, *r* = 0.21), or in the ECAS ALS-nonspecific subscore (mean 28.9 vs. 26.2; U = 424.5, Z = 1.889, *p* = 0.059, *r* = 0.23). Significance levels: * *p* ≤ 0.05, ** *p* ≤ 0.01, *** *p* ≤ 0.001. Effect sizes of r = 0.10, *r* = 0.30, and *r* = 0.50 served as thresholds for small, medium and large effects [30], respectively. Abbreviations: SMA, Spinal Muscular Atrophy; ALS, Amyotrophic Lateral Sclerosis; ECAS, Edinburgh Cognitive and Behavioural ALS Screen; max., maximal.

In the direct comparison of the cognitive profile across all SMA and ALS patients, significant differences between both groups were observed. Not only did the SMA patients show superior scores in the ECAS total score (mean 115.3 vs. 102.6; T = 3.539, df = 40.657, *p* = 0.001, *r* = 0.49) but also in a variety of subdomains (Figure 2). This included significantly higher scores in ECAS language (mean 27.3 vs. 24.6; U = 275.5, Z = −3.847, *p* < 0.001, *r* = 0.47) and ECAS executive function (mean 39.7 vs. 34.6; U = 383.5, Z = −2.394, *p* = 0.017, *r* = 0.29) leading to superiority of SMA patients in the ALS-specific subdomain (mean 86.1 vs. 76.4; U = 319.5, Z = −3.177, *p* = 0.001, *r* = 0.39). Not only ALS-specific domains showed significant superiority of SMA patients. SMA patients also reached a significantly higher mean score in ECAS memory (mean 17.5 vs. 14.7; U = 401.5, Z = −2.172, *p* = 0.030, *r* = 0.26), which is regarded as an ALS-nonspecific ECAS subdomain. The remaining subdomains did not differ statistically significantly between both groups. Regarding the WST score of SMA and ALS patients (mean 31.3 vs. 30.8), no significant difference was observed (U = 287, Z = −0.671, *p* = 0.502, *r* = 0.09).

### 3.4. Comparison of Cognitive Performance between SMA and ALS after Administration of Specifically Age- and Education-Adjusted ECAS Cut-Off Scores

According to the specifically age- and education-adjusted ECAS cut-off values [35], no SMA patient had a pathological ECAS total score. However, looking at the ECAS subdomains, SMA patients scored below the cut-off in the ALS-specific subdomain (26.5%) more often than in the ALS-nonspecific domain (5.9%). No SMA patient scored below the cut-off for the domain of verbal fluency, whereas for the domains of visuospatial and executive function, a higher number of pathological scores was detected (17.6% and 11.8%, respectively) (Table 2).

By administration of these cut-off scores to both groups, a significant difference in the number of pathological scores between SMA and ALS patients could only be detected for the ECAS total score (*n* = 0 vs. *n* = 8; 0% vs. 23.5%, *p* = 0.005) and its subdomains ECAS language (2 vs. 9; 5.9% vs. 26.5%, *p* = 0.021) and ECAS verbal fluency (0 vs. 7; 0% vs. 20.6%, *p* = 0.011). The remaining domains of the ECAS did not show any significant difference in number of pathological scores between both disease groups (Table 2).

In contrast, when comparing the number of pathological scores of SMA patients with less than 16.5 years of education (*n* = 18) and of ALS (*n* = 29), the only significant difference between both groups was found in the total ECAS total score (0 vs. 8; 0% vs. 27.6%, *p* = 0.017).

### 3.5. Influencing Factors on Cognitive Function

Neither the WST nor any cognitive domain of the ECAS correlated significantly with gender (*n* = 68) or extent of physical impairment, including ALSFRS-R score (*n* = 63), wheelchair dependence (*n* = 62), classification into a care level (corresponding to the German social insurance [32], *n* = 55) or SMA subtype (*n* = 34).

However, an earlier disease onset and longer disease duration were both strongly associated with higher scores in all of the ECAS cognitive domains, except for ECAS visuospatial ability. As shown by applying a multiple linear regression analysis, these associations were only existent due to correlation with either years of education or age of the patient at testing. Therefore, as expected and already stated by Lulé et al. in 2015, a strong correlation of age at testing and years of education with scoring higher in the ECAS could be observed [34]. Higher scores in the WST were also associated with the number of years of education (Table 3). However, when observing the association of age and cognitive scores in SMA patients only, there was no significant inverse correlation of higher age and cognition. In fact, in SMA, higher age was even associated with higher scores in WST (*p* = 0.005) and the ALS-specific subdomain of the ECAS (*p* = 0.014). An association between disease onset or disease duration with higher ECAS scores could not be observed.

## 4. Discussion

To our knowledge, this is the first multicenter study that focused on the direct comparison of the cognitive performance of SMA and ALS patients, as both patient groups suffer from a neurodegenerative disease with comparable motor function impairment. This study revealed a comparable intelligence (detected with WST) between adult SMA and ALS patients. However, SMA patients, with a disease onset in early childhood, demonstrated higher scores in distinct cognitive abilities, as assessed by ECAS. Moreover, we observed higher educational levels overall in SMA than in ALS patients.

ECAS has been developed to assess cognition in patients with ALS by taking disease-specific features such as the influence of physical disability into account. It also has a high specificity and sensitivity and strong clinical validity [34,45]. Impairment of ALS-specific (language, verbal fluency, executive function) and ALS-nonspecific cognitive functions (memory, visuospatial) can be determined using cut-off scores which are adjusted to age and education [35]. For SMA, currently no published and validated data regarding the use of ECAS are available.

In SMA patients, we found significantly higher scores in the ECAS total as well as memory, language, executive and ALS-specific domains, compared to ALS patients. Furthermore, ALS patients scored lower not only in ALS-specific cognitive domains of the ECAS compared to SMA but also in parts of the ALS-nonspecific subdomains. Therefore, one can assume a superior cognitive function in SMA independent of the cognitive impairment that may occur during the disease course of ALS. In addition, no significant differences between both groups were detected in the number of pathological scores when using age- and education-adjusted cut-off values. Therefore, our results underscore the importance of methodologically sound comparisons between groups of patients that differ regarding age and education, in particular when age-dependent cognitive functions such as memory and executive functions are considered.

Yet, the high number of SMA patients below the cut-off values suggests a possible impact of SMA on cognition, similar to the cognitive involvement in ALS. It could be demonstrated that lower age at testing correlated with better cognitive performance in both patient groups. Nevertheless, in SMA, higher age was even associated with higher scores in WST and the ALS-specific subdomain of the ECAS. Taking this into consideration, the higher average age of ALS patients compared to SMA patients, might at least partially explain that SMA patients performed better in the memory (and thus ALS-nonspecific) domain of the ECAS than ALS patients did [46]. However, using age- and education-adjusted cut-offs for ECAS [35], ALS patients scored below the cut-off more frequently than SMA patients did, mainly in the two language-related domains ECAS language and ECAS verbal fluency. Among others, these cognitive domains represent the crystalized intelligence, that is knowledge-based and education-dependent, in contrast to fluid intelligence, which corresponds to the ability to solve problems by reasoning (e.g., executive functions) [47].

Age, however, has only shown a low significant correlation with the ECAS total score and ALS-specific score in healthy controls in previous studies. Older subjects were found to perform worse than younger ones in the subdomain executive function, but this trend did not reach significance for the other subdomains or ECAS total in a study in healthy subjects in Germany [35]. The difference of age at testing between both groups in this study might, therefore, be less meaningful for interpretation of the results.

Next, a higher educational level has been identified to positively correlate with ECAS total and almost all subdomains [35]. Interestingly, educational levels in SMA were found to be significantly higher than in ALS patients, which might explain higher ECAS scores. We hypothesize that the predominantly infantile disease onset in SMA triggers higher levels of education. When physically demanding jobs (which often have lower educational requirements) are not an option, the scope of possible careers is shifted to mentally demanding jobs with higher educational requirements. This is in accordance with von Gontards hypothesis that “motor incapacitation might lead to a selective development of academic skills and verbal functions—therefore the patients appear to be more ‘intelligent’” [24]. He also stated that children suffering from SMA need the time until adolescence to compensate for their physical incapacitation through cognition [24]. In total, 33 of 34 examined SMA patients in our study developed the disease long before reaching adulthood. Therefore, as one reason why SMA patients scored significantly higher in cognitive function, early compensation of motor function impairment through cognition can be assumed. However, looking at the SMA patients’ group only, neither onset of disease nor disease duration showed any significant correlation with cognition. Hence, the hypothesis that this compensation increases with age and disease duration is not backed-up by our data. The crucial point for higher cognitive function in SMA in fact seems to be a disease onset in childhood and physical incapacitation before choosing an educational and vocational career. Nevertheless, it has to be mentioned that family background, societal aspects and access to the best suitable education most probably play important roles. Children suffering from SMA who do not receive intensive support and encouragement may not have the same possibilities to develop compensatory mechanisms or may not even thrive into adulthood at all.

While a variety of studies showed that physical activity in healthy adolescents has positive effects on cognition [48,49], physical incapacitation early in life may also lead to positive effects on cognitive function, but by other means. One explanation might be that the neurobiological critical period of adolescence is strongly affected by experience, which can influence the way in which the environment cannot only lead to susceptibility to disorders but might also offer the opportunity to change developmental paths [50]. In the case of SMA patients, different experiences in adolescence, where their physical handicap compels them to focus on cognitive abilities, may alter their developmental trajectory and result in a significantly higher level of education. In contrast to SMA patients, ALS patients in this study had frequently worked in physically demanding jobs, e.g., concrete builder, gardener, baker or electronics technician. This highlights the relevance of early support for patients with motor impairments and underlines that these patients do not inevitably have a restricted development and disadvantages regarding education and professional activities, but even confirm the opposite.

Beyond this, we observed no significant difference in scoring of the vocabulary test WST between both groups, which argues against a higher level of intelligence in SMA patients per se. As the vocabulary test is suitable to measure crystallized intelligence, it is also capable of displaying general verbal intelligence [36,37]. Thus, this is another hint on SMA patients reaching their, in comparison to ALS patients, superior cognitive function through compensation and not as an inherent characteristic of their disease. However, the WST as a vocabulary test alone is not sufficient to fully assess general intelligence in its entirety; in the future, rather an entire test battery should be used.

Surprisingly, the level of functional motor impairment measured by ALSFRS-R showed no association with the cognitive profile of patients, neither in SMA nor in ALS patients, which is in contrast to data by Mix et al. (in press) [25]. A likely explanation may be that not the current motor handicap but the level of impairment during childhood and adolescence—the time when the compensational process might have taken place—affects the cognitive profile in a patient’s adult life. Moreover, it has to be noted that Mix used another score, the Hammersmith Functional Motor Scale Expanded (HFMSE) [51,52], for measurement of physical function.

Although ALS patients with frontotemporal dementia (FTD) were excluded from the study, possible cognitive impairment in ALS patients can still be an important factor that might have contributed to the significantly higher cognitive test results of SMA patients. Previous studies reported incidences of FTD from about 12.6% to 15% in ALS and some kind of cognitive impairment in general in up to 50% of ALS patients [7,8,9]. The influence of ALS-related cognitive impairment on the results of this study may be discussed controversially. The number of pathological scores between both groups only differed in ECAS total score, in language and verbal fluency, representing the domains associated with the clinical stage as defined by the King’s staging system in ALS [53]. Therefore, an influence of ALS-related cognitive impairment has to be taken into account. On the other hand, no difference in the remaining ECAS domains could be seen. Mix et al. showed that the cognitive function in SMA is no worse than in healthy subjects (in press) [25]. Therefore, differences in cognitive performance between both groups may not only be explained by ALS-related cognitive impairment but may additionally be attributed to the above-mentioned compensatory developments in childhood in the SMA group.

Comparison of our results of cognitive performance in SMA with other neuromuscular conditions such as Duchenne muscular dystrophy (DMD) is not fully accurate, as in previous studies, not adults but mostly children were examined, and other cognitive measures than the ECAS and WST were used [19,20,21,22,23]. Previous studies in SMA- vs. DMD-affected children and adolescents showed inconsistent results of, on the one hand, similar cognitive abilities of SMA compared to DMD [18,19], and, on the other hand, superior abilities in SMA [20,21,22,23]. It has been assumed that especially education-dependent aspects of intelligence (i.e., (id est), crystalized aspects of intelligence) might be higher in patients with SMA, while the “general” intelligence of SMA children seemed to be comparable to age-matched healthy controls [24].

Interpretation of our data is certainly limited, as the ECAS has not been validated in SMA and only a cross-sectional analysis was carried out. Furthermore, SMA and ALS differ regarding age at onset, duration of illness and speed of functional decline. One reason why comparing patients with ALS to those with SMA is, however, useful, is the opportunity to rule out the impact of motor symptoms as a potential confounding factor in ALS. Low performance in typical tasks can be due to an actual cognitive deficit, but also due to motor dysfunction (e.g., slowed speech motor skills) or a combination of both factors. When ALS patients are compared to patients with similar motor symptoms (e.g., SMA patients), the interpretation of cognitive test results becomes more valid. Thus, a strategy to solve the validity problem is to compare diseases with similar non-cognitive (e.g., motor) symptoms. Our study may be viewed as being a paradigmatic validation study that reveals cognitive deficits in ALS patients when they are compared to SMA patients, who show similar motor impairments.

Another limitation is that the vocabulary test WST as a measure for the general level of intelligence was not assessed in all ALS patients and moreover, would be more meaningful as part of a comprehensive test battery. Third, our SMA and ALS volunteers were recruited in specialized NMD clinics. Thus, we cannot exclude a selection bias towards well-educated patients who are more likely to be referred to the specialized centers. Therefore, our sample may not be a perfect representation of larger SMA and ALS populations, but we assume that our cohorts are still representative for a subset of adults who are willing and able to participate in future clinical trials. Regarding potential gender effects, there is a high amount of evidence for gender similarity in most, but not all, psychological variables [54]. Moreover, the gender distribution did not differ significantly within our patient cohorts, so that we did not expect any influence anyway. Another question is the pathological significance of our observations, since most of the presented differences are small despite being statistically significant. Of course, this cannot definitely be answered, but also former studies showed that small but statistically significant differences in the ECAS of ALS patients compared to healthy controls were detectable [34]. Data suggest that there is a correlation of cognitive performance measured by ECAS with structural disease pathology which further indicates clinical significance of our results [55,56]. A further limitation of this study is caused by the small sample size. However, ALS and SMA are rare diseases. Especially SMA patients more frequently consulted specialized therapy centers since the availability of nusinersen treatment in 2017 [14]. Furthermore, SMA is mainly a disease with onset in early childhood and rapid progression frequently resulting in death in childhood or adolescence. Therefore, larger datasets from adult SMA patients are still lacking. This natural disease history course is currently changing with the approval of new disease modifying therapies so that more data will be available in the future [57]. Overall, our study findings should be confirmed in larger patient cohorts, since we only examined a small number of cases with an exploratory study purpose. Thus, we hope that our pilot study may inspire researchers in the field to further investigate these important aspects of cognitive abilities in SMA.

## 5. Conclusions

In conclusion, our study showed a comparable intelligence in the WST vocabulary test in SMA and ALS. Nevertheless, we found a better cognitive performance in SMA patients in comparison to those suffering from ALS in subdomains of the ECAS. No connection of cognitive scores with motor skills per se could be shown. A compensational improvement of mainly knowledge-based, education-dependent crystallized cognitive functions due to a stronger focus on education as a consequence of severe physical impairment in childhood and adolescence is the most suitable explanation.

It is important to state that the cognitive abilities acquired in childhood and adolescence are still present in adult SMA patients. Therefore, severe motor impairments do not inevitably lead to disadvantages in educational and professional careers, but early educational support seems to be able to stimulate a compensatory development. Our results may, therefore, further encourage SMA children and their families.

Future longitudinal multicenter studies should further elucidate disease-related aspects of cognitive functions in SMA and additional influencing factors.

## Figures and Tables

**Table 1:** Clinical characterization of spinal muscular atrophy (SMA) and amyotrophic lateral sclerosis (ALS) patients. This table shows disease factors with a possible influence on cognitive function. Effect sizes of *r* = 0.10, *r* = 0.30, and *r* = 0.50 served as thresholds for small, medium and large effects [30], respectively. Abbreviations: SMA, Spinal Muscular Atrophy; ALS, Amyotrophic Lateral Sclerosis; ALSFRS-R, Amyotrophic Lateral Sclerosis Functional Rating Scale revised form; SD, standard deviation; *n*, number; vs., versus.

	SMA (*n* = 34)	ALS (*n* = 34)	SMA vs. ALS
Age *n* (years)	34	34	
MEDIAN (RANGE)	38.5 (19–64)	66.0 (46–79)	
MEAN (SD)	40.2 (13.1)	65.8 (9.4)	
			T = −9.253, df = 59.734; *p* < 0.001; *r* = 0.77
Gender *n*	34	34	
female	11 (32.4%)	13 (38.2%)	
			U = 544.00; Z = −0.504; *p* = 0.614; *r* = 0.06
SMA type *n*	34		
type 1	0		
type 2	14 (41.2%)		
type 3	19 (55.9%)		
type 4	1 (2.9%)		
Disease onset *n* (at age in years)	34	32	
MEDIAN (RANGE)	1.8 (0–47)	63.5 (41–77)	
MEAN (SD)	6.5 (9.3)	63.4 (9.4)	
			U = 2.00; Z = −6.957; *p* < 0.001; *r* = 0.85
Disease duration *n* (years)	34	33	
MEDIAN (RANGE)	32.5 (3–62)	1.4 (0–18)	
MEAN (SD)	33.8 (13.2)	2.2 (3.2)	
			U = 9.50; Z = −6.920; *p* < 0.001; *r* = 0.85
Years of education *n*	34	34	
MEDIAN (RANGE)	16.0 (10–24)	12.5 (8–21)	
MEAN (SD)	16.3 (3.5)	13.1 (2.8)	
			U = 272.00; Z = −3.762; *p* < 0.001; *r* = 0.46
Education level *n*	34	19	
Intensified secondary education *(German Abitur)*	21 (61.8%)	6 (31.6%)	
Extensive secondary education *(German Realschule)*	10 (29.4%)	4 (21.1%)	
Basic secondary education *(German Hauptschule)*	2 (5.9%)	9 (47.4%)	
Other	1 (2.9%)	0 (0%)	
ALSFRS-R *n*	34	29	
MEDIAN (RANGE)	30.0 (10–48)	33.0 (16–41)	
MEAN (SD)	30.8 (10.8)	31.3 (7.0)	
			U = 493.00; Z = 0.000; *p* > 0.999; *r* = 0.00
Ambulatory status *n*	34	27	
ambulatory	9 (26.5%)	16 (59.3%)	
manual/electric wheelchair use (part-time, always)	25 (73.5%)	11 (40.7%)	
Care level ^1^ *n*	32	23	
0	11 (34.4%)	6 (26.1%)	
1	1 (3.1%)	6 (26.1%)	
2	0 (0%)	5 (21.7%)	
3	5 (15.6%)	4 (17.4%)	
4	9 (28.1%)	1 (4.3%)	
5	6 (18.8%)	1 (4.3%)	

^1^ Care level: classification at the care level according to the German health care system, with higher levels indicating a greater loss of autonomy and a need for more individual support (care level 1 = minor impairment of individual autonomy, care level 5 = severe impairment of individual autonomy with special care requirements); also see [31,32].

**Table 2:** Age- and education-adjusted ECAS cot-off scores. Number of SMA and ALS patients who reached ECAS under cut-off scores (with respect to the total score and ECAS subdomains). Abbreviations: SMA, Spinal Muscular Atrophy; ALS, Amyotrophic Lateral Sclerosis; ECAS, Edinburgh Cognitive and Behavioural ALS Screen; *n*, number; vs., versus; OR, odds ratio.

	SMA	ALS	SMA vs. ALS
ECAS total *n*	34	34	
ECAS total under cut-off *n*	0	8	
Percentage	0%	23.5%	
			*p* = 0.005 ^1^; OR = 0.00
ECAS memory *n*	34	34	
ECAS memory under cut-off *n*	2	6	
Percentage	5.9%	17.6%	
			*p* = 0.259 ^1^; OR = 0.29
ECAS visuospatial *n*	34	34	
ECAS visuospatial under cut-off *n*	6	9	
Percentage	17.6%	26.5%	
			χ²(1) = 0.770; *p* = 0.380; OR = 0.60
ECAS ALS-nonspecific *n*	34	34	
ECAS ALS-nonspecific under cut-off *n*	2	6	
Percentage	5.9%	17.6%	
			*p* = 0.259 ^1^; OR = 0.29
ECAS language *n*	34	34	
ECAS language under cut-off *n*	2	9	
Percentage	5.9%	26.5%	
			χ²(1) = 5.134; *p* = 0.021; OR = 0.17
ECAS verbal fluency *n*	33	34	
ECAS verbal fluency under cut-off *n*	0	7	
Percentage	0%	20.6%	
			*p* = 0.011 ^1^; OR = 0.00
ECAS executive *n*	34	34	
ECAS executive under cut-off *n*	4	7	
Percentage	11.8%	20.6%	
			χ²(1) = 0.976; *p* = 0.323; OR = 0.51
ECAS ALS-specific *n*	34	34	
ECAS ALS-specific under cut-off *n*	9	6	
Percentage	26.5%	17.6%	
			χ²(1) = 0.770; *p* = 0.380; OR = 1.68

^1^ Fisher’s Exact Test.

**Table 3:** Results of multiple linear regression analysis and influencing factors on cognitive test performance. Disease (SMA or ALS), disease onset, disease duration, years of education and age were identified as the factors that had an influence on the different cognitive tests/domains. However, after using multiple linear regression, it emerged that only years of education and age at testing showed a significant correlation with test performance. Only significant scores are shown for better overview. Abbreviations: ECAS, Edinburgh Cognitive and Behavioural ALS Screen; WST, Wortschatztest.

	ECAS Total	ECAS Memory	ECAS Visuospatial	ECAS ALS Nonspecific	ECAS Language	ECAS Verbal Fluency	ECAS Executive	ECAS ALS Specific	WST
Disease									
r	−0.399	−0.276		−0.256	0.438	−0.264	−0.361	−0.386	
*p*	0.001	0.023		0.035	0.000	0.031	0.002	0.001	
Disease onset									
r	−0.394	−0.314		−0.291	−0.406		−0.372	−0.363	
*p*	0.001	0.010		0.018	0.001		0.002	0.003	
Disease duration									
r	0.315				0.333	0.245	0.320	0.338	
*p*	0.009				0.006	0.047	0.008	0.005	
Years of education									
r	0.439	0.282		0.246	0.440	0.344	0.421	0.431	0.615
*p*	<0.001	0.020		0.043	<0.001	0.004	<0.001	<0.001	<0.001
Age									
r	−0.345	−0.392		−0.383	−0.332		−0.308	−0.274	
*p*	0.004	0.001		0.001	0.006		0.011	−0.024	
After multiple linear regression	Only years of education significant (b = 1.470; *p* = 0.011)	Only age significant (b = −0.147; *p* = 0.024)		Only age significant (b = −0.142; *p* = 0.009)	Only years of education significant (b = 0.247; *p* = 0.016)	Only years of ducation ignificant (b = 0.379; *p* = 0.045)	Only years of education significant (b = 0.678; *p* = 0.011)	Only years of education significant (b = 1.163; *p* = 0.013)	
